# Galanin Coordinates Macrophage-Associated Fibro-Inflammatory Response and Mitochondrial Integrity in Myocardial Infarction Reperfusion Injury

**DOI:** 10.3390/ijms25116211

**Published:** 2024-06-05

**Authors:** Lesia Savchenko, Solomiia Kramar, Nika Todua, Dimitri Marsal, Ryeonshi Kang, Audrey Swiader, Nathalie Pizzinat, Oksana Kunduzova

**Affiliations:** 1 Department of Internal Medicine, Poltava State Medical University, 23 Shevchenko, 36000 Poltava, Ukraine; lesia.savchenko@inserm.fr; 2 National Institute of Health and Medical Research (INSERM) U1297, Paul Sabatier University, Cedex4, 31432 Toulouse, France; solomiia.kramar@inserm.fr (S.K.); nika.todua@yahoo.com (N.T.); dimitri.marsal@inserm.fr (D.M.); ryeonshi.kang@inserm.fr (R.K.); audrey.swiader@inserm.fr (A.S.); nathalie.pizzinat@inserm.fr (N.P.); 3 Histology and Embryology Department, I. Horbachevsky Ternopil National Medical University, 46001 Ternopil, Ukraine

**Keywords:** inflammation, cardiac remodeling, mitochondria, apoptosis, fibrosis

## Abstract

Myocardial infarction activates an intense fibro-inflammatory reaction that is essential for cardiac remodeling and heart failure (HF). Bioactive peptide galanin plays a critical role in regulating cardiovascular homeostasis; however, its specific functional relevance in post-infarction fibro-inflammatory reprogramming remains obscure. Here, we show that galanin coordinates the fibro-inflammatory trajectory and mitochondrial integrity in post-infarction reperfusion injury. Aberrant deposition of collagen was associated with a marked increase in CD68-positive macrophage infiltration in cardiac tissue in mice subjected to myocardial ischemia/reperfusion (I/R) for 14 days compared to sham controls. Furthermore, we found that the myocardial expression level of a specific marker of M2 macrophages, CD206, was significantly down-regulated in I/R-challenged mice. In contrast, galanin treatment started during the reperfusion phase blunted the fibro-inflammatory responses and promoted the expression of CD206 in I/R-remodeled hearts. In addition, we found that the anti-apoptotic and anti-hypertrophic effects of galanin were associated with the preservation of mitochondrial integrity and promotion of mitochondrial biogenesis. These findings depict galanin as a key arbitrator of fibro-inflammatory responses to cardiac I/R injury and offer a promising therapeutic trajectory for the treatment of post-infarct cardiovascular complications.

## 1. Introduction

Myocardial infarction (MI) is the major cause of global non-transmittable deaths and a primary contributor to heart failure (HF) [1]. The limited regenerative capacity of the heart culminates in a massive loss of myocytes and abnormal deposition of extracellular matrix (ECM) components under MI conditions, which is called cardiac remodeling. Myocardial fibrosis is a principal element of the cardiac remodeling process, dictating HF manifestations [2]. Although the reperfusion phase can reverse myocardial remodeling, the incidence and amplitude of cardiac fibrosis can dynamically progress [3]. In patients with post-infarction left ventricular (LV) dysfunction, fibrotic tissue remodeling increases and deteriorates cardiac function [2]. In clinical practice, the occurrence of the cardiac fibrosis stage is one of the main prognostic factors in heart disease [4].

Myocardial fibrosis matures in a milieu of inflammatory cell infiltration and the degree of immune cell infiltration is directly associated with the severity of the tissue fibrosis [5]. Clinical and experimental observations have indicated that the accumulation of cardiac macrophages, the most abundant immune cells, correlates with the severity of the tissue fibrosis and cardiac injury [6]. The infiltration of inflammatory cells leads to cardiac fibrosis in murine models of cardiac remodeling [7]. The myocardial expression of tissue-resident macrophages correlates significantly with the progression of interstitial fibrosis in human heart disease [8]. Moreover, the selective depletion of macrophages reduces myocardial fibrosis [9]. These studies support a decisive role for fibro-inflammatory responses in the progression of cardiac injury and dysfunction.

Recent studies have provided evidence that bioactive peptides coordinate crucial regulatory functions in fibro-inflammatory processes. Galanin is a 29/30-amino-acid-long bioactive peptide that belongs to the galanin family and forms the galaninergic system. The galaninergic system is a specific signaling system that controls neuromodulation and neurotransmission and was firstly uncovered by Tatemoto et al. [10]. Galanin is widely distributed in the central and peripheral nervous tissues in various species, with roles in numerous biological processes including sleep, nociception, depression, memory, feeding, anxiety, metabolism, and stress [11,12]. Studies by Kofler et al. [13] showed that galanin is involved in skin immunity, as its expression was demonstrated to be upregulated in inflammation-remodeled tissue. In poststroke inflammation, galanin regulates the release of inflammatory cytokines and inflammatory cell infiltration in the brain [14]. Galanin regulates cellular responses by activating three distinct G-protein-coupled receptors, galanin receptor 1 (GalR1), GalR2, and GalR3, which diverge in their distributions, pharmacology, and signaling. The different galanin receptors are coupled to a variety of signal transduction pathways, with both GalR1 and GalR3 acting predominantly on the inhibitory G_i_ proteins, while GalR2 can activate either stimulatory G_q_ proteins or inhibitory G_i_ proteins [15]. Recently, we demonstrated that in the heart, GalR2 is the predominant receptor subtype that transduces the effects of galanin in cardiac cells [16]. In response to myocardial injury, the galaninergic system coordinates antioxidant and metabolic activities that may result in a powerful cardioprotective effect on the failing heart [17]. Decrypting the cues that initiate galanin-mediated action in cardiac tissue is critical for the development of therapeutic strategies to modulate the fibro-inflammatory responses in failing hearts. 

In this study, we evaluated whether galanin affects the cardiac fibrotic remodeling associated with macrophage-related inflammatory motifs in I/R-challenged hearts. We found that prolonged galanin treatment blunts the I/R-mediated fibro-inflammatory trajectory and preserves mitochondrial integrity in response to cardiac I/R injury.

## 2. Results

**Figure 1:**
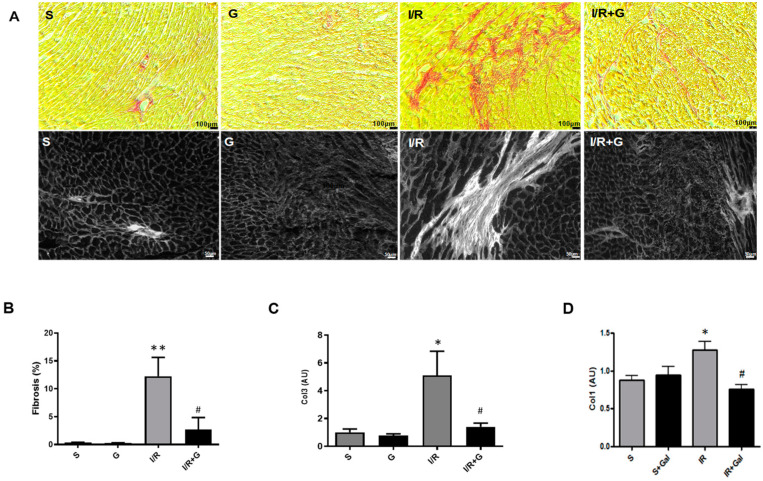
Galanin blunts cardiac fibrosis in myocardial infarction reperfusion injury. (**A**) Representative images of cardiac sections stained with Sirius Red (top panel; collagen fibers: red color) and visualized by polarized light microscopy (bottom panel; collagen fibers: white color) from mice subjected to sham operation or 30 min of cardiac ischemia and 14 days of reperfusion (I/R). (**B**) Quantification of myocardial fibrosis in (**A**). (**C**,**D**) Myocardial mRNA levels of collagen 1 (Col 1) and collagen 3 (Col 3) in vehicle- or galanin-treated (G) mice after 14 days of I/R injury, *n* = 5. Data presented as the mean ± SEM. * *p* < 0.05, ** *p* < 0.01 vs. sham, # *p* < 0.05 vs. I/R according to one-way ANOVA followed by Bonferroni’s post hoc test.

Progressive structural modifications associated with fibro-inflammatory motifs of the myocardium is a major characteristic feature of failing hearts [18,19]. We first investigated whether galanin affects I/R-induced myocardial fibro-inflammatory responses after myocardial damage in mice. An excessive accumulation of fibrillar collagen in the myocardium was detected in mice subjected to 14 days of I/R injury using Sirius Red staining and histomorphological examination by polarized light microscopy (Figure 1). In contrast, the galanin post-ischemic treatment markedly decreased myocardial fibrosis in I/R-challenged mice compared with vehicle-treated mice (Figure 1A,B). Consistent with these observations, in galanin-treated I/R mice, we found a significant decrease in the mRNA expression of collagen type I and type III in the heart (Figure 1C,D).

**Figure 2:**
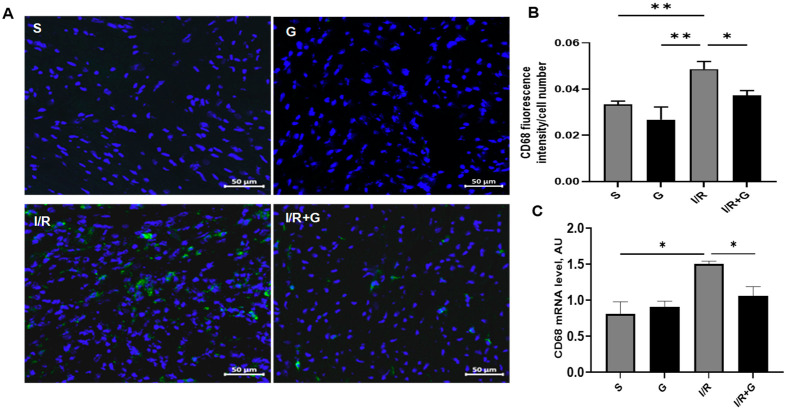
Galanin inhibits macrophage-associated inflammatory reactions in myocardial infarction reperfusion injury. (**A**) CD68 immunostaining (green fluorescence) was performed to detect inflammatory cell populations in cardiac sections from vehicle- or galanin-treated mice subjected to 30 min of cardiac ischemia and 14 days of reperfusion. Nuclei were counterstained with DAPI (blue). (**B**) Quantification of CD68-positive cells from (**A**), *n =* 5. (**C**) Myocardial CD68 mRNA levels in vehicle- or galanin-treated mice subjected to I/R or sham, *n =* 5. Data presented as the mean ± SEM. * *p* < 0.05, ** *p* < 0.01 according to one-way ANOVA followed by Bonferroni’s post hoc test.

A recent report suggested that macrophages promote the transition from myocardial I/R injury to cardiac fibrosis in mice [20]. To evaluate the effects of galanin in macrophage-associated inflammatory responses to I/R injury, we next examined the myocardial expression of CD68, a macrophage marker, in vehicle- or galanin-treated I/R-challenged hearts. As shown in Figure 2A,B, I/R injury induced the expression of CD68 in cardiac tissue, demonstrating a high macrophage number in the damaged areas of the myocardium compared to the control sham group. 

Immunofluorescence staining identified the massive presence of CD68-positive cells in the infarct and border territory of the left ventricle. However, chronic treatment with galanin blunted the I/R-mediated increase in the number of CD68-positive cells, indicating that galanin suppresses cardiac macrophage-associated inflammatory responses in the post-ischemic myocardium. The analysis of mRNA expression levels demonstrated that the treatment with galanin depressed the I/R-stimulated CD68 expression levels in mice (Figure 2C).

**Figure 3:**
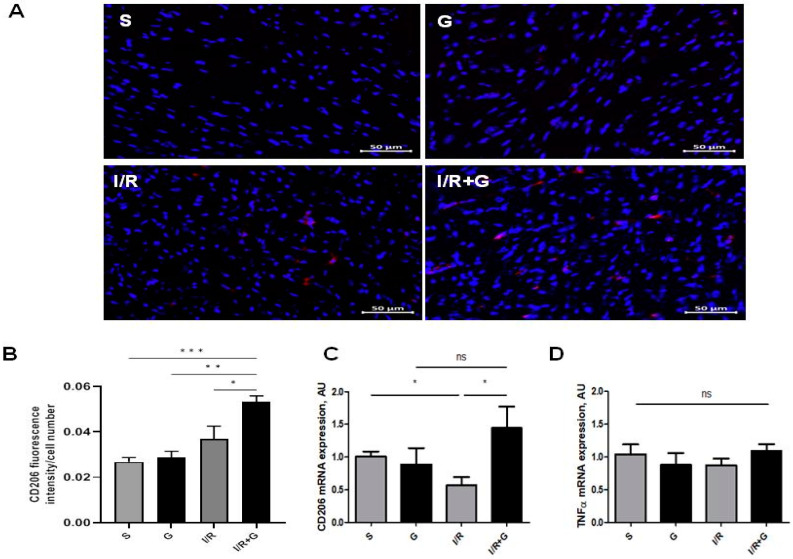
Galanin promotes the anti-inflammatory trajectory in I/R-remodeled hearts. Mice were exposed to 30 min of cardiac ischemia and 14 days of reperfusion. (**A**,**B**) Representative images and quantification of CD206 (red) in vehicle- or galanin-treated mice subjected to I/R damage, *n* = 4. Nuclei were counterstained with DAPI (blue). (**C**,**D**) Myocardial mRNA levels of CD206 and TNFα in vehicle- or galanin-treated control sham and I/R-challenged mice, *n* = 5. Data presented as the mean ± SEM; ns: not significant. * *p* < 0.05, ** *p* < 0.01, *** *p* < 0.001, according to one-way ANOVA followed by Bonferroni’s post hoc test.

We next characterized M2-phenotype macrophages by studying the mRNA and protein abundance of CD206 in cardiac tissue from vehicle- or galanin-treated mice subjected to 14 days of I/R injury. The immunohistochemical staining showed no significant changes between the sham and I/R groups after 14 days of cardiac I/R injury (Figure 3A,B). However, we found that CD206 was highly expressed in cardiac sections from galanin-treated I/R mice compared to the vehicle-treated I/R group or sham mice (Figure 3A,B). A comparative analysis of mRNA expression levels demonstrated that I/R induces a down-regulation of the myocardial expression of CD206 compared to the control group, which was prevented by galanin treatment in mice (Figure 3C). In contrast, tumor necrosis factor α (TNF*α*) mRNA levels showed no significant differences between the experimental groups after 14 days of I/R injury compared to the vehicle-treated group (Figure 3D). 

**Figure 4:**
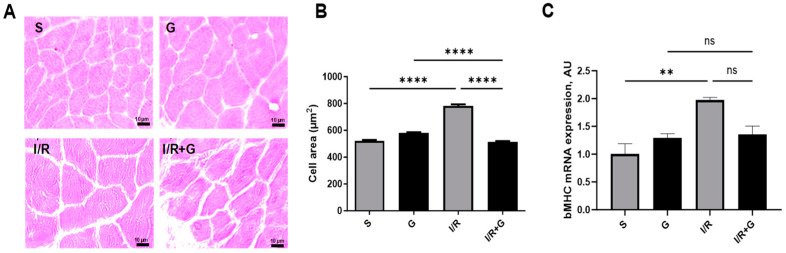
Galanin reduces cardiac hypertrophy in myocardial infarction reperfusion injury. (**A**) Representative photomicrographs of cardiac tissue sections with H&E staining and (**B**) quantitative analysis of the cross-sectional area. (**C**) Myocardial mRNA levels of β-MHC in vehicle- or galanin-treated control sham and I/R-challenged mice, *n* = 5. Data presented as the mean ± SEM; ns: not significant. ** *p* < 0.01, **** *p *< 0.001 according to one-way ANOVA followed by Bonferroni’s post hoc test.

We next evaluated whether galanin counteracts cardiac hypertrophy and apoptosis in response to I/R injury. As shown in Figure 4A,C, I/R-challenged hearts displayed characteristics of pronounced cardiac hypertrophy as evidenced by an increased myocyte cross sectional area and by elevated β-myosin heavy chain (β-MHC) expression patterns. However, we found a significantly decreased myocyte cross-sectional area and β-MHC mRNA expression in the hearts of galanin-treated mice compared to vehicle-treated mice after 14 days of I/R injury. 

**Figure 5:**
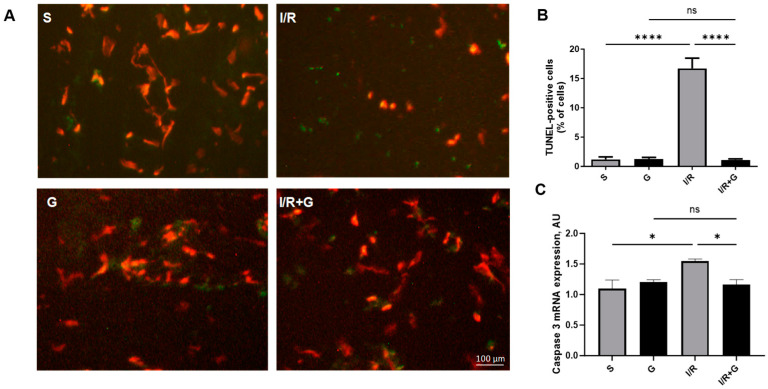
Galanin prevents myocardial apoptosis in I/R-challenged hearts. (**A**) Representative photomicrographs of TUNEL staining of cardiac sections from vehicle- or galanin-treated mice exposed to 30 min of cardiac ischemia and 14 days of reperfusion. The green cells indicate the TUNEL-positive apoptotic cells. (B) Quantification of TUNEL-positive cells from (A), *n *= 5. (**C**) Myocardial mRNA levels (**C**) of caspase 3 in vehicle- or galanin-treated mice exposed to I/R injury. Data presented as the mean ± SEM; ns: not significant. * p < 0.05, **** p < 0.0001 according to one-way ANOVA followed by Bonferroni’s post hoc test.

To detect cardiac apoptosis triggered by I/R injury, we next performed terminal deoxynucleotidyl transferase dUTP nick end labeling (TUNEL) staining in vehicle- or galanin-treated mice after 14 days of I/R injury. We demonstrated that mice exposed to cardiac I/R injury for 14 days exhibited a significant increase in TUNEL-positive apoptotic cells compared to the control sham group (Figure 5A,B). In addition, I/R injury markedly elevated caspase-3 expression in I/R-challenged hearts. Importantly, compared to vehicle-treated mice, galanin markedly reduced the I/R-mediated increase in TUNEL-positive apoptotic cells and myocardial caspase-3 expression 14 days after I/R damage.

**Figure 6:**
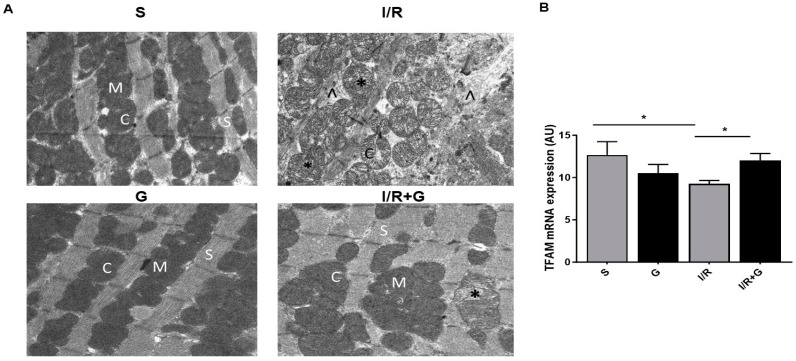
Galanin preserves mitochondrial integrity in myocardial infarction reperfusion injury. (**A**) Electron micrographs of hearts from vehicle- or galanin (G)-treated mice subjected to cardiac I/R injury. Cardiac tissues were harvested for examination of the myocardial ultrastructure by transmission electron microscopy. Magnification: 14,000×. (M)—mitochondria; (C)—cristae; (S)—sarcomere; (*)—damaged mitochondria; (∧)—disrupted myofibrils. (**B**) Myocardial TFAM mRNA levels in vehicle- or galanin-treated mice subjected to I/R or sham. *n* = 5. Data are expressed as the mean ± SEM. * p < 0.05 according to one-way ANOVA followed by Bonferroni’s post hoc test.

Mitochondrial dysfunction is a primary signature of failing hearts [21]. We further evaluated the effects of galanin in I/R-triggered alterations in mitochondrial architecture after 14 days of cardiac I/R injury. As shown in Figure 6A, cardiac mitochondria from the post-ischemic myocardium were characterized by increased mitochondrial clumping, swelling, and disrupted cristae, reflecting mitochondrial ultrastuctural disorganization in the infarct border zone. Furthermore, I/R-challenged hearts showed interrupted sarcomeres with the fragmentation of myofibrils and damaged Z lines. In contrast, treatment with galanin preserved mitochondrial integrity and the initial sarcomere structure with intact myofibrils and conserved Z lines in I/R-challenged hearts compared to vehicle-treated I/R hearts. In addition, the treatment with galanin increased the myocardial expression of mitochondrial transcription factor A (TFAM), a master regulator of mitochondrial biogenesis, compared with vehicle-treated mice (Figure 6B). 

**Figure 7:**
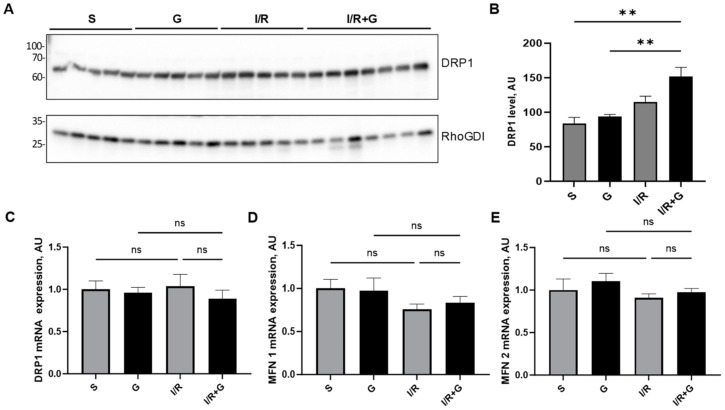
Galanin promotes mitochondrial fission in I/R-remodeled hearts. (**A**) Representative Western blot of DRP1 protein expression and (B) quantification of (**A**). Myocardial mRNA levels of (**C**) DRP1, (**D**) MFN1, and (**E**) MFN2 in vehicle- or galanin-treated control sham and I/R-challenged mice, *n* = 5. Mice were exposed to 30 min of cardiac ischemia and 14 days of reperfusion. Data presented as the mean ± SEM; ns: not significant. ** *p* < 0.01 according to one-way ANOVA followed by Bonferroni’s post hoc test.

Mitochondria are dynamic organelles that continually undergo cycles of fission and fusion. To examine mitochondrial fission, we next evaluated the myocardial expression of dynamin-related protein 1 (DRP1) in cardiac tissue 14 days post-infarction. The cardiac DRP1 expression pattern showed no significant differences between the control sham and I/R groups (Figure 7A,B). In contrast, the treatment of mice with galanin increased DRP1 protein expression compared to the sham controls (Figure 7A,B). In addition to DRP1 myocardial expression, we evaluated the expression of mitofusin-1 (MFN1) and mitofusin-2 (MFN2), the arbitrators of mitochondrial fusion. The analysis of their mRNA expression levels demonstrated no significant differences in the mRNA expression of DRP1, MFN1, and MFN2 between the experimental groups (Figure 7C–E).

## 3. Discussion

The accumulating evidence indicates that post-infarction fibrotic and inflammatory remodeling of cardiac tissue is mechanistically linked to compromised mitochondrial function. In this study, we found that prolonged treatment with galanin, starting during the reperfusion phase, blunts the macrophage-associated fibro-inflammatory motif of I/R injury and preserves the mitochondrial integrity of the myocardium. In a mouse model of post-infarct reperfusion damage, we demonstrated that galanin attenuated myocardial abnormalities in response to I/R insult by (1) inhibiting the aberrant deposition of collagen fibers and macrophage infiltration; (2) promoting the M2 macrophage anti-inflammatory phenotype; (3) reducing myocardial apoptosis and hypertrophy; and (4) preserving mitochondrial ultrastructural assembly. 

The amplitude of cell death of the post-infarct myocardium caused by I/R injury has been shown to be associated with the severity of cardiac dysfunction as well as mortality [22]. Therefore, interventions to reduce cardiac cell death have been investigated in pre-clinical and clinical studies [23]. Previous studies demonstrated that decreased mortality and improved ventricular function are associated with a reduction in apoptotic cell death [24]. In this study, we demonstrated that prolonged treatment with galanin prevents myocardial apoptosis caused by I/R damage, suggesting a regulatory role of this peptide in programmed cell death reprogramming of the myocardium. Recently, we demonstrated that galanin can suppress apoptotic cell death in failing hearts through the activation of the FoxO1 pathway [16]. We found that FoxO1 suppression counteracts galanin-mediated anti-apoptotic activity, indicating that galanin regulates the apoptotic status of cardiac cells through a FoxO1-dependent mechanism. Since myocardial apoptosis calibrates cardiac performance, a galanin-mediated reduction in the number of apoptotic cells can provide a benefit for the preservation of ventricular function. The accurate evaluation of cardiac functional parameters is necessary for the assessment of galanin’s effects on the long-term progression of left ventricular dysfunction.

We found also that I/R induced cardiomyocyte hypertrophy, which was characterized by an increase in the cardiomyocyte surface area and elevated expression of the marker gene β-MHC at 14 days after injury. In contrast, the galanin treatment prevented hypertrophy in I/R-challenged hearts, suggesting a central role of galanin in the coordination of the hypertrophic responses to I/R damage. 

The inflammatory motif is a significant component in the progression of cardiac I/R injury [9]. In response to myocardial I/R injury, macrophages, immune cells that are widely distributed in cardiac tissues, rapidly enter the area of the lesions and release effector factors, initiating the inflammatory reaction [6]. Macrophages represent a central component of the first-line immune riposte against cardiac I/R damage [9]. In a pathological scenario, macrophages display two phenotypes including a pro-inflammatory M1-like motif or anti-inflammatory M2-like phenotype [6,9]. Tissue macrophages have specific gene expression patterns and distinct sets of transcription factors that calibrate their properties and functions [25]. The pro-inflammatory M1 subtype is characterized by the expression of CD80, CD86, and CD16/32 and the release of TNF-α and other pro-inflammatory cytokines involved in tissue injury, whereas M2 macrophages are anti-inflammatory and overexpress arginase-1 (Arg-1), CD206 (mannose receptor), and interleukin-10 (IL-10) [25]. This balance between the M1 and M2 phenotypes determines the cell/organ fate in inflammation or injury [26]. Our results clearly demonstrated that galanin reversed the cardiac I/R-induced decrease in the myocardial expression of CD206, suggesting a role of this peptide in macrophage reprogramming towards the M2-like phenotype. Interestingly, the galanin-dependent promotion of the M2-like phenotype was associated with a marked reduction in cardiac fibrosis. Since macrophages calibrate the fibro-inflammatory responses in cardiac remodeling, galanin’s control over the large-scale expression of specific markers that distinguish the phenotypes of macrophages during I/R injury needs further studies. 

Ischemia and post-ischemic reperfusion cause a wide array of functional and ultrastructural alterations in mitochondria [21]. Mitochondria are pivotal organelles that are responsible for multiple vital cell functions, including respiration, oxidative phosphorylation, and regulation of apoptosis [27]. The burst of post-ischemic ROS can dissipate the adenosine triphosphate (ATP) cell potential and induce mitochondrial swelling, which subsequently leads to an activation of the apoptotic pathway and mitochondrial abnormalities [25]. In this study, we demonstrated the protective action of prolonged galanin treatment on cardiac mitochondrial integrity during I/R. We found that galanin prevented mitochondrial disorganization including mitochondrial swelling and disruption of the mitochondrial cristae that could contribute to a reduction in post-ischemic structural and metabolic abnormalities. Mitochondria sustain normal vital functions by maintaining a balanced interplay between two contradictory events, fission and fusion, to retain a mitochondrial network. Fusion and fission events occurring concurrently determine the architecture and size of the mitochondria by regulating mitochondrial dynamics [27]. Mitochondrial fusion is the process of merging of two mitochondria to produce a distinctly healthy mitochondrion. In contrast, the process by which a single mitochondrion may divide into two or more daughter organelles to remove damaged and fragmented mitochondria is referred to as mitochondrial fission [27]. Therefore, the myocardium needs highly sophisticated, balanced control mechanisms for mitochondrial dynamics and mitochondrial quality-control mechanisms to conserve the fitness of mitochondrial pools and networks. The present study demonstrated that galanin induces DRP1 expression in I/R-challenged hearts, suggesting that the galaninergic system contributes to the regulation of mitochondrial fission. These data are in line with a previous study that demonstrated that DRP1 mediates mitochondrial autophagy and protects the heart against energy stress [28]. These findings clearly pinpointed that the disruption of DRP1 induces mitochondrial elongation, autophagy suppression, and mitochondrial dysfunction, thereby promoting cardiac dysfunction and increased susceptibility to ischemia and reperfusion [28]. Future research will hopefully shed light on the role of galanin receptors in DRP1 activity and mitochondrial function in failing hearts. 

Recently, we revealed that in the heart and cultured cardiomyocytes, GalR2 is the predominant receptor subtype that orchestrates the functional trajectory of galanin [16]. In response to myocardial injury, the galaninergic system controls the antioxidant and metabolic status of the heart and may contribute to a cardioprotective effect in the failing myocardium [17]. Decrypting the galaninergic system’s role in cardiac tissue is crucial for the development of effective therapeutic strategies to regulate fibro-inflammatory reactions in failing hearts. 

Taken together, this study demonstrated that prolonged treatment with galanin, starting during the reperfusion phase, blunts macrophage-associated fibro-inflammatory motifs and preserves mitochondrial integrity in left ventricular remodeling post-myocardial infarction. We also showed that galanin prevents apoptotic cell death and promotes the polarization of macrophages towards the M2-like phenotype in the post-ischemic myocardium. These data suggest a promising strategy for ameliorating the consequences of coronary disease. 

## 4. Materials and Methods

### 4.1. Animals

Animal experiments were performed in accordance with the guidelines established by the European Communities Council Directive (2010/63/EU Council Directive Decree) and approved by the local Centre National de la Recherche Scientific ethics committee.

Male C57Bl/6J mice (three months old) were purchased from the Janvier Labs, (Le Genest-Saint-Isle, France) and maintained in a temperature-controlled room (25 °C) with a natural day/night cycle and fed a standard chow diet and given ad libitum access to water. The animals were subjected to a sham operation (S) or cardiac ischemia/reperfusion (I/R) for 14 days.

Animals were randomly divided into 4 groups: (1) sham + PBS (S) group (*n* = 5); (2) sham + galanin (G) group (*n* = 5); (3) ischemia/reperfusion + PBS (I/R) group (*n* = 5); and (4) ischemia/reperfusion + galanin (I/R + G) group (*n* = 5).

The mice then received, for 14 consecutive days, intraperitoneal injections of vehicle (PBS) or galanin (5 mg/kg/day) in a final volume of 100 μL. Treatment with galanin started after 15 min of reperfusion. The mice intraperitoneally received vehicle or galanin every 24 h for 14 days.

### 4.2. Experimental Protocol

A mouse model of I/R was used as previously described [16]. The mice were intubated and placed under mechanical ventilation after undergoing general anesthesia with an intraperitoneal injection of ketamine (125 mg/kg) and xylazine (10 mg/kg). A left parasternotomy was performed to expose the hearts, and a 0.4 mm polyethylene suture was placed around the left anterior descending coronary artery. A snare was placed on the suture, and regional myocardial ischemia was produced by tightening the snare. After 30 min of ischemia, the occlusive snare was released to initiate reperfusion. Sham-operated control mice underwent the same surgical procedures except that the snare was not tightened.

### 4.3. Immunofluorescence and Histological Studies

Heart tissues were embedded in optimal cutting temperature compound (OCT) (Sigma-Aldrich, Saint-Quentin-Fallavier, France) under ice-cold 2-methylbutane. For CD68 staining, frozen sections (10 µm) were fixed in 4% paraformaldehyde, followed by permeabilization and blocking in PBS with 3% bovine serum albumin and 0.1% Triton X-100 at room temperature. The sections were immunostained overnight with anti-CD68 (MCA1957GA) or CD206 (MR5D3) followed by secondary Alexa fluor antibodies (Molecular Probes, Invitrogen, Cergy-Pontoise, France). Images were acquired using a Zeiss LSM 900 confocal microscope and ZEN blue 3.2 image analysis software (Zeiss, Marly le roi, France). Four representative assessment zones were established in relation to the infracted area: two peri-infarct zones, one zone in the middle of the infarct, and a zone in the posterior segment of the interventricular septum representing a non-infarcted control area of the tissue slice. Immunofluorescence staining for CD68 or CD206 was evaluated by quantification of fluorescence intensity normalized to the number of nuclei (DAPI-positive cells) per field of view using the ImageJ software version 1.54i. Hematoxylin–eosin (H&E) and Sirius Red staining were performed on 10 μm heart cryosections as previously described [17]. The extent of cardiac fibrosis was quantified using ImageJ software.

### 4.4. Transmission Electron Microscopy 

Ultrastructural studies of cardiac tissue by electron microscopy were performed as previously described [16]. Briefly, the cardiac tissues were fixed in cold 2.5% glutaraldehyde/1% paraformaldehyde, post-fixed in 2% osmium tetroxide, embedded in resin, and sectioned. 

### 4.5. Western Blot

Extraction of proteins from cardiac tissues was performed as previously described [29]. In short, we used RIPA buffer for protein extraction, and the lysates were quantified after clarification using the Bio-Rad Protein Assay (Bio-Rad, Hercules, CA, USA). Proteins were resolved via SDS-PAGE and Western blotting. Immunoreactive bands were detected via chemiluminescence using the Clarity Western ECL Substrate (Bio-Rad, Hercules, CA, USA) on a ChemiDoc MP Acquisition system (Bio-Rad, Hercules, CA, USA). The antibodies used in this study were DRP1 (sc-32898) and RhoGDI (sc-365190) from Santa Cruz Biotechnology (Santa Cruz, CA, USA). RhoGDI was used as a loading control.

### 4.6. Quantitative RT-PCR Analysis

Total RNA was isolated from mouse hearts using the GenElute Mammalian Total RNA Miniprep Kit (Sigma Aldrich, Saint-Quentin-Fallavier, France). The total RNA (500 ng) was reverse transcribed using a High-Capacity cDNA Reverse Transcription Kit (Applied Biosystems, Villebon sur Yvette, France) in the presence of random hexamers. Real-time quantitative PCR was performed as previously described [18]. The apical region of the heart was used for RNA extraction. Primer sequences are detailed in Table 1. The expression of the target gene was normalized to Rplp0(36B4) or GAPDH expression.

### 4.7. Statistical Analysis

Statistical comparison between groups was performed using one-way ANOVA followed by Bonferroni’s post hoc test using GraphPad Prism version 9.00 (GraphPad Software, Inc. (San Diego, CA, USA). Data are expressed as mean ± SEM.

## 5. Conclusions

The bioactive peptide galanin provides cardioprotection by reducing the macrophage-associated fibro-inflammatory responses to myocardial I/R injury and preserving mitochondrial integrity in mice. These findings provide important information regarding the cardiac benefits of galanin in a pathological scenario of cardiac remodeling processes and offer a new perspective for tackling aberrant fibro-inflammatory responses to I/R injury.

## Figures and Tables

**Table 1:** Real-time qPCR primer sequences.

Gene	Forward Sequence (5′-3′)	Reverse Sequence (5′-3′)
*Caspase 3*	GACTGATGAGGAGATGGCTTG	TGCAAAGGGACTGGATGAAC
*β MHC*	TGAGCCTTGGATTCTCAAACGT	AGGTGGCTCCGAGAAAGGAA
*CD68*	GCCCAAGGAACAGAGGAAGACT	GTAGGGCTGGCTGTGCTTTCT
*CD206*	ATGCCAAGTGGGAAAATCTG	TGTAGCAGTGGCCTGCATAG
*Collagen type I*	TGTGTGCGATGACGTGCAAT	GGGTCCCTCGACTCCTACA
*Collagen type III*	AAGGCGAATTCAAGGCTGAA	TGTGTTTAGTACAGCCATCCTCTAGAA
*DRP1*	CAGATATGGCAACGTCAGAGG	AACCCTTCCCATCAATACATCC
*GAPDH*	CTTTGTCAAGCTCATTTCCTGG	TCTTGCTCAGTGTCCTTGC
*MFN1*	GACAGCCCAGGTACAGATG	AGCCGCTCATTCACCTTATG
*MFN2*	GGATGACCTCGTGCTGATG	AACTGCTTCTCCGTCTGC
*Rplp0(36B4)*	TGACATCGTCTTTAAACCCCG	TGTCTGCTCCCACAATGAAG
*TFAM*	GCACCCTGCAGAGTGTTCAA	CGCCCAGGCCTCTACCTT
*TNFα*	TGGGACAGTGACCTGGACTGT	TTCGGAAAGCCCATTTGAGT

## Data Availability

The data presented in this study are available on request from the corresponding author.
